# Microglial Transient Receptor Potential Melastatin 2 Deficiency Accelerates Seizure Development via Increasing AMPAR‐Mediated Neuronal Excitability

**DOI:** 10.1002/mco2.70271

**Published:** 2025-07-14

**Authors:** Yingwei Xu, Luyu Ye, Zhisheng Li, Yi Zhang, Ning Hua, Xiaojun Wang, Wangjialu Lu, Jing Xi, Liying Chen, Cenglin Xu, Jiajia Fang, Jianhong Luo, Linhua Jiang, Feng Han, Zhong Chen, Yi Wang, Wei Yang

**Affiliations:** ^1^ Institute of Pharmacology & Toxicology NHC and CAMS Key Laboratory of Medical Neurobiology College of Pharmaceutical Sciences School of Medicine Zhejiang University Hangzhou China; ^2^ Zhejiang Key Laboratory of Neuropsychopharmacology School of Pharmaceutical Sciences Zhejiang Chinese Medical University Hangzhou China; ^3^ Department of Biophysics and Department of Neurology the Fourth Affiliated Hospital Zhejiang University School of Medicine Yiwu China; ^4^ Department of Neurobiology Affiliated Mental Health Center College of Brain Science and Brain Medicine School of Medicine Zhejiang University Hangzhou China; ^5^ Sino‐UK Joint Laboratory of Brain Function and Injury of Henan Province and Department of Physiology and Pathophysiology Xinxiang Medical University Xinxiang China; ^6^ International Joint Laboratory for Drug Target of Critical Illnesses School of Pharmacy Nanjing Medical University Nanjing China

**Keywords:** TRPM2 channel, seizure, microglia, AMPAR, neuron

## Abstract

Epilepsy is one of the most common neurological disorders, characterized by the enhancement of neural excitability from a neurocentric perspective. Emerging evidence indicates that microglia play a pivotal role in the pathogenesis of epilepsy through complex and various mechanisms that is still not fully understood. In this study, we demonstrate that the deficiency of transient receptor potential melastatin 2 (TRPM2) channel, a calcium‐permeable nonselective cation channel, significantly accelerates seizure development in multiple mouse seizure models, including MES‐ and pentylenetetrazole(PTZ)‐induced seizure model, intrahippocampal KA model, hippocampal kindling model, without affecting seizure susceptibility in initial acute seizure. Notably, it is the deficiency of TRPM2 specifically in microglia, rather than in CaMKIIα^+^ excitatory neurons or PV^+^ interneurons, that primarily responsible for seizure development. Moreover, microglial TRPM2 deficiency increases the excitability of hippocampal pyramidal neurons by enhancing the AMPAR‐mediated excitatory synaptic transmission independent of changes in the expression of inflammatory cytokines. These findings reveal a previously unrecognized, inflammation‐independent mechanism by which microglial instead of neuronal TRPM2 channel contributes to seizure development, highlighting microglial TRPM2 as a novel potential therapeutic target for epilepsy by specifically targeting microglial TRPM2 channel.

## Graphical abstract

The deficiency of transient receptor potential melastatin type 2 (TRPM2) channel as a calcium permeable nonselective cation channel, accelerates seizure development in various mouse seizure models including PTZ‐, KA‐, and kindling‐induced models, but does not affect seizure susceptibility in initial acute seizure. Interestingly, the deficiency of TRPM2 in microglia, but not CaMKIIα^+^ or PV^+^ neurons, is mainly responsible for seizure development. Moreover, microglial TRPM2 deficiency increases the excitability of hippocampal pyramidal neurons via enhancing the AMPAR‐mediated excitatory synaptic transmission but does not influence the expression of inflammation cytokines.

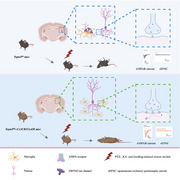

## 1 Introduction

Epilepsy is one of the prevalent neurological disorders, characterized by recurrent spontaneous seizures resulting from excessive or hypersynchronous neuronal activity and affects approximately 65 million people worldwide [1, 2]. Traditionally, epileptic seizures are widely regarded to occur as a result of an enhancement of neural excitability due to the excitatory–inhibitory imbalance [3]. Neural excitability is tightly regulated by ion channels, such as sodium (Na^+^), calcium (Ca^2+^), and potassium (K^+^) channels, as well as synaptic transmission, particularly the balance between excitatory glutamatergic and inhibitory GABAergic neurotransmission [4, 5, 6]. Gene mutations related to these channels or key targets in synaptic transmission are central to the pathophysiology of epileptic seizures [7, 8]. Antiseizure drugs (ASDs) are main treatments for epilepsy; however, current ASDs are only effective on control of seizure, but do not show benefit for seizure development or epileptogenesis. Moreover, approximately one of third people fail to achieve seizure control [9], leading to enormous burden to patients and society. Thus, it is critical to identify the pathogenesis of epilepsy to search new insight for treatment.

Tomography studies have shown that the human brain contains equal numbers of neurons and glial cells [10] and glia/neuron ratio varies uniformly across different brain structures of mammalian species [11]. Moreover, glia cells can modulate survival and activity of neurons [12]. Recently, accumulating investigations show that microglia, which are highly adaptable glial cells, have been involved in various nervous system disease including the epilepsy [13, 14, 15, 16]. Studies have shown that microglial morphological changes and immunoreactivity are concomitant with the process of epilepsy in rodents, also microglia are activated in patients of different types of epilepsy [17, 18, 19]. The mechanism of microglia involved in epilepsy usually focus on in inflammation‐related function; however, the accurate role of microglia in epilepsy is still debatable. For example, both the proinflammatory cytokines including IL‐1β and TNF‐α and anti‐inflammatory cytokines such as IL‐4 and IL‐10, all increased in the development of seizure, suggesting a complex role of microglia in epilepsy [20]. Besides, in the epileptic brain, microglia can regulate the activity of neurons by synaptic pruning to induce the imbalance of hippocampal synaptic excitatory/inhibitory transmission [21]. In addition, Eyo et al. [22] found that deficiency of extracellular Ca^2+^ can increase the interaction of microglia and neurons during epilepsy. All of these indicate that microglia may display an indispensable role in the initiation and development of seizure.

The transient receptor potential melastatin 2 (TRPM2) channel is a Ca^2+^ permeable, nonselective cation channel mediating cellular response to various stimuli, including reactive oxygen/nitrogen species (ROS/RNS), ADP‐ribose (ADPR), and several ADPR analogues [23]. Accumulating evidence including our previous studies have indicated that TRPM2 is involved in many ROS‐related diseases such as inflammation, ischemia–reperfusion injury, stroke, diabetes, pericytes injury, neurodegeneration, and depression [24, 25, 26, 27, 28]. Accordingly, emerging evidence showed that ROS is elevated after seizure and plays a key role in prolonged seizure [29] and status epilepticus (SE) [30, 31]; however, the underlying mechanism remains elusive.

In our study, we first evaluate the seizure behavior of TRPM2 deficiency mice in multiple mouse seizure models, our results showed TRPM2 deletion consistently accelerates seizure development in these models. To further determine the contribution of TRPM2 in certain cell type, we constructed the *Trpm2^fl/fl^* mice with loxP sites flanked on either side of exon 4 of TRPM2 channel. Our results showed that selectively removed TRPM2 in either CaMKIIα^+^ or PV^+^ neurons did not affect seizure development, while microglial TRPM2 deletion accelerates seizure development. Moreover, our data showed that the relative mRNA levels of hippocampal IL‐1β, IL‐6, IL‐4, IL‐10, and TGFβ in seizure mice were not changed after microglial TRPM2 deletion, suggesting that microglial TRPM2 mediated seizure development independent on inflammation. Finally, we found that microglial TRPM2 deficiency increases excitatory synaptic transmission by enhancing the AMPAR‐mediated current without influencing the inhibitory synaptic transmission, which results in the elevated pyramidal neuronal excitability. Our findings for the first time reveal microglial TRPM2 regulate the seizure development in an inflammation‐independent way and provide a new insight of epilepsy therapy.

## 2 Results

### 2.1 TRPM2 Deficiency Accelerates Seizure Development in Mouse Seizure Models

**FIGURE 1:**
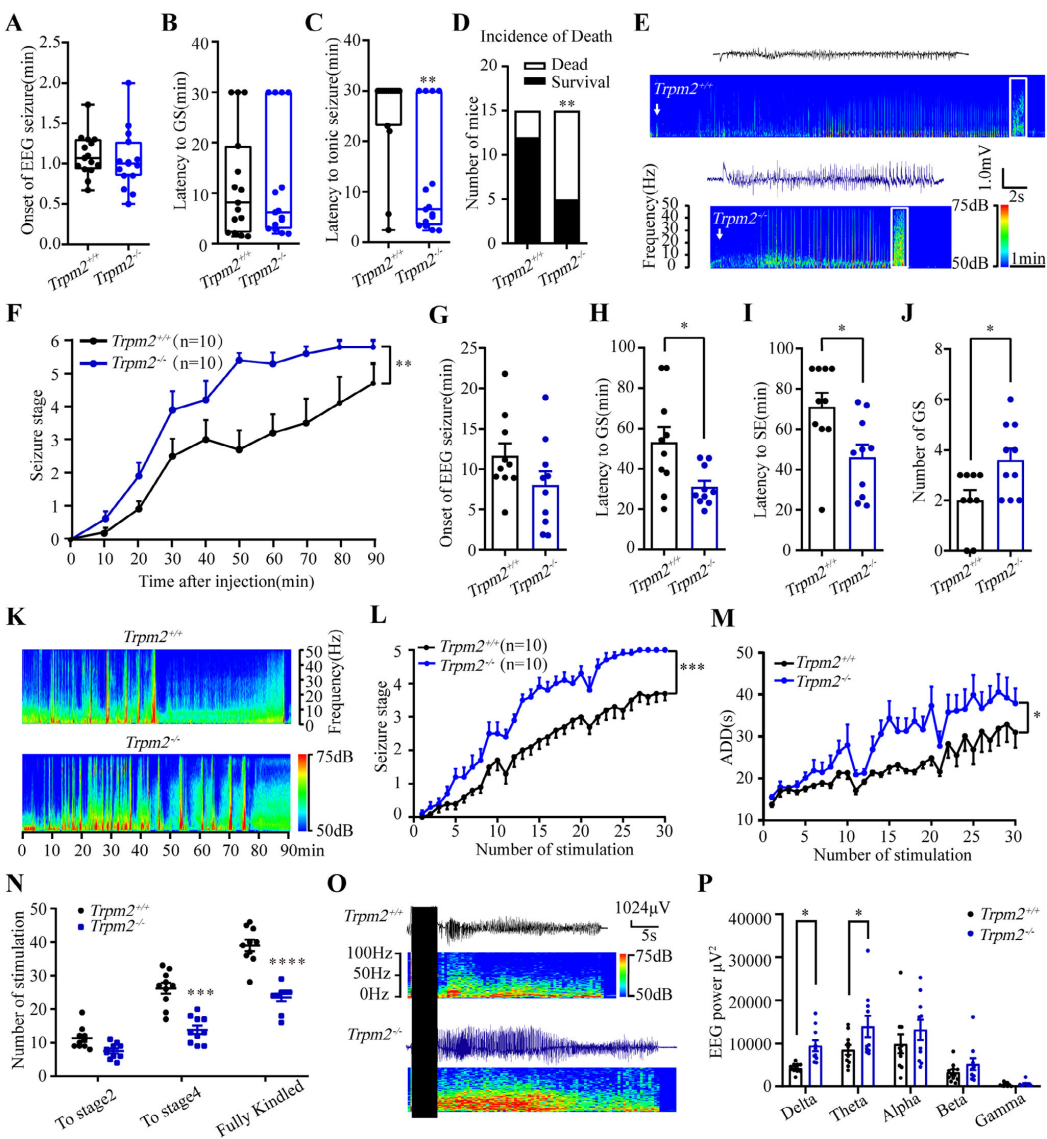
TRPM2 deficiency accelerates seizure development in a variety of mouse seizure models. (A–D) Effects of TRPM2 channel deficiency on onset of EEG seizure (A), latency to GS (B), latency to tonic seizure (C), and incidence of death (D) in PTZ‐induced seizure model (*n* = 15 for each group). (E) Representative EEGs and corresponding energy spectra of seizure activity in PTZ‐induced seizure model. The arrows represent the time for PTZ injection. (F–K) Effects of TRPM2 channel knockout on seizure stage (F), onset of EEG seizure (G), latency to GS (H), latency to SE (I), number of GS (J) in intrahippocampal KA model (*n* = 10 for each group). (K) Representative EEGs and corresponding energy spectra of seizure activity in intrahippocampal KA model. (L–P) Effects of TRPM2 channel knockout on the development of seizure stage (L), ADD (M), number of stimulations needed to each seizure stage (N) in hippocampal kindling model (*n* = 10 for each group). (O and P). Representative EEGs, corresponding EEG spectra power (O), and quantification of EEG power (P) recorded from CA3 during the fully kindled state. Error bars are means ± s.e.m.; Mann–Whitney test was used for *Trpm2^+/+^* versus *Trpm2^−/−^* in A‐Cd; Chi‐square test was used for *Trpm2^+/+^* versus *Trpm2^−/^* ^−^ in D; two‐way ANOVA test was used for *Trpm2^+/+^* versus *Trpm2^−/−^* in F; two‐tailed unpaired *t*‐test was used for *Trpm2^+/+^* versus *Trpm2^−/−^* in G–J; align‐and‐rank data for a nonparametric ANOVA was used for *Trpm2^+/+^* versus *Trpm2^−/−^* in L; general linear model with repeated measures was used for *Trpm2^+/+^* versus *Trpm2^−/−^* in M; and two way ANOVA test was used for *Trpm2^+/+^* versus *Trpm2^−/−^* in N, P. *, **, ***, and **** represent *p *< 0.05, 0.01, 0.001, and 0.0001, respectively.

To investigate the role of TRPM2 channel in epilepsy, we first evaluated the seizure behaviors of *Trpm2^−/^* ^−^ mice in different mouse seizure models, including MES‐ and pentylenetetrazole (PTZ)‐induced seizure model, intrahippocampal KA model, and hippocampal kindling model. As shown in Figure 1A–D, we found that in PTZ‐induced seizure model, the onset of EEG seizure (Figure 1A) and the latency to GS (Figure 1B) had no significant change, but the latency to tonic seizure (Figure 1C) was significantly decreased and the incidence rate of death (Figure 1D) was increased in *Trpm2^−/−^* mice, compared with these in *Trpm2^+/+^* mice. The representative EEG and corresponding energy spectra of seizure activity measured in *Trpm2^+/+^* and *Trpm2^−/−^* mice (Figure 1E) indicated the proseizure effect of *Trpm2* deletion. In intrahippocampal KA model, the seizure stage (Figure 1F) demonstrated that the development of epilepsy of *Trpm2^−/−^* mice was significantly faster than *Trpm2^+/+^* mice. Specifically, compared with *Trpm2^+/+^* mice, the onset of EEG seizures (Figure 1G) had no change, but the latency to GS (Figure 1H) and SE (Figure 1I) were shortened; the number of GS (Figure 1J) was increased in *Trpm2^−/−^* mice. The representative EEG and corresponding energy spectra of seizure activity (Figure 1K) demonstrated that the deficiency of TRPM2 channel aggravated the severity of KA‐induced seizures. However, we found that compared with *Trpm2^+/+^* mice, the threshold and tonic duration of *Trpm2^−/−^* mice were not changed in MES‐induced acute seizure model (Figure S1). The results showed that deficiency of TRPM2 channel accelerated seizure development, but not affected seizure susceptibility in initial acute seizure.

Next, we evaluated whether *Trpm2* deletion exert any proseizure effect on a hippocampal kindling model that mimics clinically complex partial seizure with generalized seizure (GS) [32]. As shown in Figure 1M–P, compared with *Trpm2^+/+^* mice, *Trpm2* deletion accelerated the progression of seizure stages behavior (Figure 1L) and prolonged the after‐discharge durations (ADDs) (Figure 1M). The proseizure effect of *Trpm2* deletion took place mainly later stages instead of the initial of seizure, as it significantly decreased the number of stimulations required to reach stage 4 and fully kindled, instead of stage 2 (Figure 1N). Consistent with behavioral observations, EEG and energy spectrum analyses indicated that *Trpm2* deletion aggravated seizure severity in kindled mice (Figure 1O,P).

To rule out the developmental influence of transgenic mice on seizure, we administered selective TRPM2 channel antagonist A23 [33] to investigate whether inhibition of TRPM2 channel could also accelerate seizure in PTZ‐induced seizure model. Similar with the seizure behavior of *Trpm2^−/−^* mice, pharmacological inhibition of TRPM2 channel by antagonist A23 did not affect the onset of EEG seizure and latency to GS (Figure S2A,B), while led to a decreased latency to tonic seizure (Figure S2C) and an increased incidence of death (Figure S2D), suggesting the important role of TRPM2 channel involved in epilepsy.

### 2.2 Selective Deficiency of TRPM2 Channel in CaMKIIα^+^ Excitatory Neurons and PV^+^ Interneurons have no Influence in Seizure Development

**FIGURE 2:**
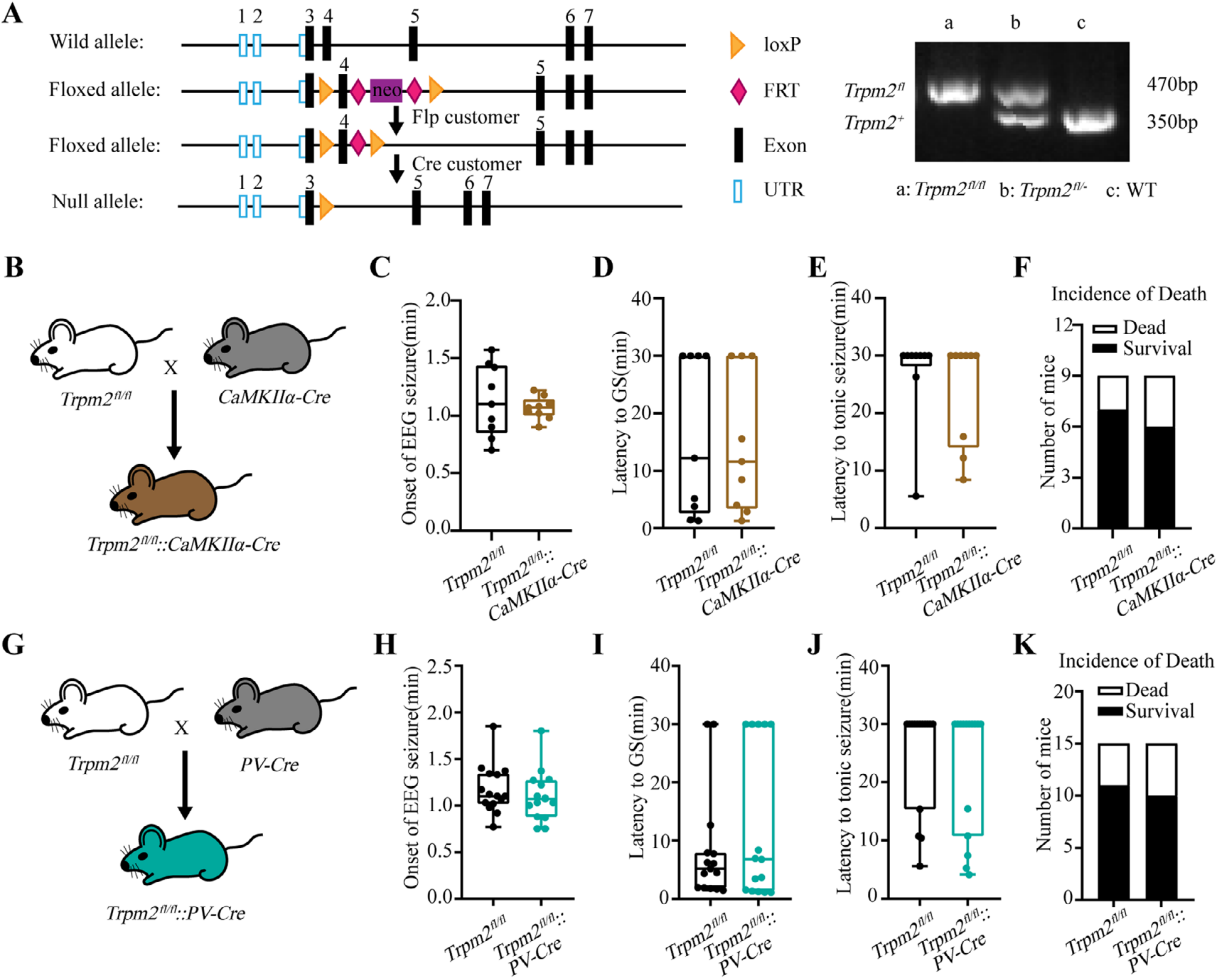
Selective deficiency of TRPM2 channel in CaMKIIα^+^ and PV^+^ neurons have no influence in seizure susceptibility. (A) Schema of *flox* gene for knockin (left), PCR analysis of the *flox* gene in mouse genome (right). (B) Selective knockout of TRPM2 channel in CaMKIIα^+^ neurons was achieved by breeding *CaMKIIα‐Cre* mice to *Trpm2^fl/fl^* mice to generate *Trpm2^fl/fl^::CaMKII‐Cre* mice (brown). (C–F) Effects of selective knockout of TRPM2 channel in CaMKIIα^+^ neurons on onset of EEG seizure (C), latency to GS (D), latency to tonic seizure (E), and incidence of death (F) in PTZ‐induced seizure model (*n* = 9 for each group). (G) Selective knockout of TRPM2 channel in PV^+^ neurons was achieved by breeding *PV‐Cre* mice to *Trpm2^fl/fl^* mice to generate *Trpm2^fl/fl^::PV‐Cre* mice (blue). (H–K) Effects of selective knockout of TRPM2 channel in PV neurons on onset of EEG seizure (H), latency to GS (I), latency to tonic seizure (J), and incidence of death (K) in PTZ‐induced seizure model (*n* = 15 for each group). Error bars are means ± s.e.m.; two‐tailed unpaired *t*‐test was used in C and H; Mann–Whitney test was used in D, E, I, and J; Chi‐square test was used in F and K.

Considering the widespread expression of TRPM2 channel in the brain, both in neurons and microglia, to investigate the cell‐specific mechanism of TRPM2 channel in epilepsy, we construct *Trpm2^fl/fl^* mice (Figure 2A), then we breed *Trpm2^fl/fl^* mice with *CaMKIIα‐Cre* mice to selectively delete *Trpm2* in CaMKIIα^+^ excitatory neurons (Figure 2B). The results in PTZ‐induced seizure model showed that the deficiency of TRPM2 channel in CaMKIIα^+^ excitatory neurons had no effect on seizure behavior (Figure 2C–F), including the onset of EEG seizures, the latency to GS and tonic seizure, and the incidence of death. As hippocampus is a key region in epileptogenesis, to further define the role of TRPM2 channel in hippocampal CaMKIIα^+^ excitatory neurons, we injected virus AAV2/9–mCaMKIIa–H2B–eGFP–P2A–iCre–WPRE–pA bilaterally into the hippocampus in *Trpm2^fl/fl^* mice to delete *Trpm2* in hippocampal CaMKIIα^+^ excitatory neurons (Figure S3A). In PTZ‐induced seizure model, the result indicated that the TRPM2 channel in hippocampal CaMKIIα^+^ excitatory neurons were not involved in seizure behavior (Figure S3B–E).

As the research demonstrated TRPM2 channel in PV^+^ interneurons participated in the firing of PV^+^ neurons [24], which may influence the seizure susceptibility via inhibitory output to excitatory neurons, we breed *Trpm2^fl/fl^* mice with *PV‐Cre* mice to selectively delete *Trpm2* in PV^+^ interneurons (Figure 2G). However, the results in PTZ‐induced seizure model (Figure 2H–K) showed that the deficiency of TRPM2 channel in PV^+^ interneurons still had no effect on seizure behavior. To further definite the role of TRPM2 channel in hippocampal PV^+^ interneurons, we injected virus rAAV–PV–CRE–EGFP–bGH polyA bilaterally into the hippocampus in *Trpm2^fl/fl^* mice to delete *Trpm2* in hippocampal PV^+^ interneurons (Figure S3F). As shown in Figure S3G–J, we found that the TRPM2 channel in hippocampal PV^+^ interneurons were not involved in seizure behavior. Moreover, in our previous work, we show that substantia nigra (SNr) plays a key role in seizure spread, especially seizure spread [34]. Meanwhile, TRPM2 is also highly expressed in this region and modulate firing pattern of PV neurons [24]. Thus, we also injected virus rAAV–PV–CRE–EGFP–bGH polyA bilaterally into the SNr in *Trpm2^fl/fl^* mice to delete *Trpm2* in SNr PV^+^ neurons (Figure S3K). As shown in Figure S3L–O, we found that the TRPM2 channel in SNr PV^+^ neurons were not involved in seizure behavior. Above results demonstrated that selective deficiency of TRPM2 channel in CaMKIIα^+^ and PV^+^ neurons, two main glutamatergic and GABAergic neurons in the forebrain, have no influence in seizure behavior.

### 2.3 Deficiency of TRPM2 Channel in Microglia Accelerates Seizure Development

**FIGURE 3:**
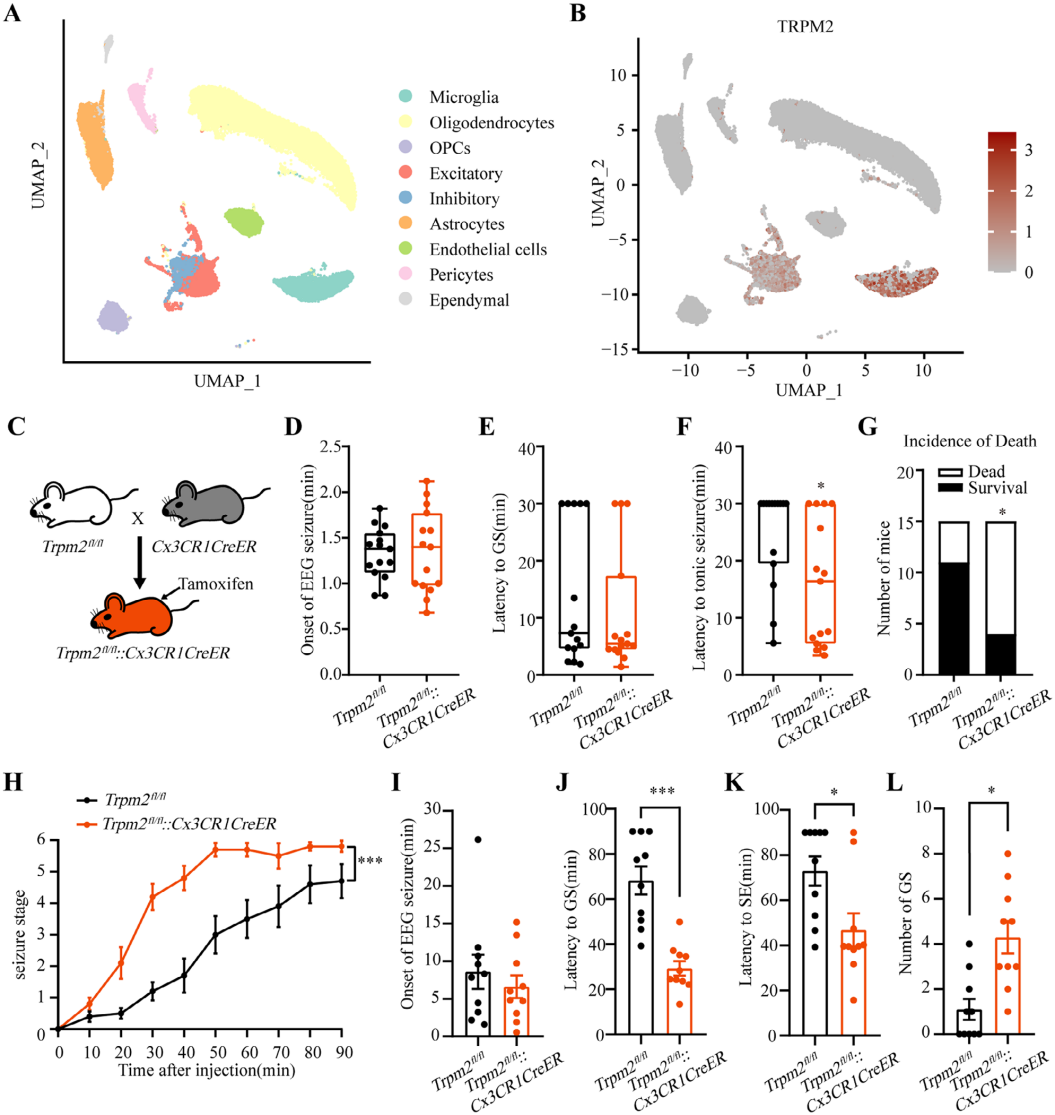
Selective deficiency of TRPM2 channel in microglia accelerates seizure development. (A) Uniform manifold approximation and projection (UMAP) plot of snRNA‐seq of human brain nuclei. The general identity of each cell cluster is annotated to the right and the percentage of each cell cluster per total cell number is indicated. (B) Normalized expression of TRPM2 is color coded and projected on the UMAP plot. (C) Selective knockout of TRPM2 channel in microglia was achieved by breeding *Cx3CR1CreER* mice to *Trpm2^fl/fl^* mice to generate *Trpm2^fl/fl^::Cx3CR1CreER* mic*e* (mandarin). (D–G) Effects of TRPM2 channel knockout in microglia on onset of EEG seizure (D), latency to GS (E), latency to tonic seizure (F), and incidence of death (G) in PTZ‐induced seizure model (*n* = 15 for each group). (H–L) Effects of selective knockout of TRPM2 channel in microglia on seizure stage (H), onset of EEG seizure (I), latency to GS (J), latency to SE (K), number of GS (L) in intrahippocampal KA model (*n* = 10 for each group). Error bars are means ± s.e.m.; two‐tailed unpaired *t*‐test was used in D and I–L; Mann–Whitney test was used in E and F; Chi‐square test was used in G; two‐way ANOVA test was used in H; * and *** represent *p *< 0.05 and 0.001, respectively.

TRPM2 channel is known to be widely distributed in the central nervous system, both in neurons and microglia, and microglial TRPM2 channel had been reported in other neurological diseases [26, 35]. In our work, reanalysis of published single‐nucleus transcriptome sequencing (snRNA‐seq) data [36] showed TRPM2 channel is highly expressed in microglia (Figure 3A,B), highlighting a key role of microglial TRPM2 channel. Then, we breed *Trpm2^fl/fl^* mice with *Cx3CR1CreER* mice to delete *Trpm2* in microglia by intraperitoneal injecting tamoxifen to induce the expression of cre‐recombinase, while the *Trpm2^fl/fl^* mice receiving tamoxifen served as control (Figure 3C). We found that the proseizure behavior of *Trpm2^fl/fl^::Cx3CR1CreER* mice were similar with *Trpm2^−/−^* mice both in PTZ‐induced seizure model and intrahippocampal KA model. Consistent with shortening the latency to tonic seizure and increasing mortality in *Trpm2^−/−^* mice, deficiency of TRPM2 channel in microglia shortened the latency to tonic seizure and increased the incidence of death (Figure 3F,G) and had no effect on the onset of EEG seizures and latency to GS (Figure 3D,E). Similarly, in intrahippocampal KA model, compared with *Trpm2^fl/fl^* mice, microglial *Trpm2* deletion accelerates seizure development (Figure 3H), shortening the latency to GS and SE (Figure 3J,K) and increasing the number of GS (Figure 3L). These results showed the importance of microglial TRPM2 channel in seizure; the proepileptic role of *Trpm2* deletion was mainly caused by the functional change of microglia. To determine the knockout efficiency of the Cre‐loxp system, we used qPCR to verify the relative mRNA expression in different conditional knockout mice. In HEK 293 cells transfected with exon4 null‐trpm2 plasmids, the TRPM2 channel currents were nearly disappeared and correspondingly the mRNA expression was null (Figure S4A,B). And the relative mRNA expression was indeed decreased in all these three mice (Figure S4C–E).

The hippocampus is a representative landmark structure for its physiological and diagnostic relevance in epilepsy [37]; it is critical to definite the effect of *Trpm2* deletion on hippocampal microglia after seizure. We found that compared with mice of sham group, the microglia were obviously activated both in the CA1 and CA3, and the deficiency of TRPM2 channel can inhibit the increased area of Iba1^+^ microglia in *Trpm2^−/−^* mice after fully kindled (Figure S5A–D), suggesting that the deficiency of TRPM2 channel inhibits the morphological activation of microglia in hippocampus. To further confirm the role of TRPM2 channel in microglial activation, we also evaluated the area of Iba1^+^ microglia in *Trpm2^fl/fl^::Cx3CR1CreER* mice after PTZ‐induced seizure, the result showed that morphological activation of microglia was inhibited after microglial *Trpm2* deletion (Figure S5E,F). Moreover, it is known that the active microglia can releases cytokines to influence neuroinflammation [38, 39]. Thus, we next tested the change fold of relative mRNA expression of proinflammation cytokines, IL‐1β, IL‐6, TNF‐α, and IFN‐γ, and anti‐inflammation cytokines, IL‐4, IL‐10, TGF‐β, and Arg1. As shown in Figure S6, surprisingly, the microglial *Trpm2* deletion had no change in cytokine release in basal states, and the expression of relative mRNA of IL‐1β, IL‐6, IL‐4, IL‐10, and TGF‐β was still elevated after microglial *Trpm2* deletion, further suggesting the release of inflammation cytokines was independent with microglial TRPM2 channel.

### 2.4 Microglial *Trpm2* Deletion Increased the Excitability of Hippocampal Pyramidal Neurons via Enhancing AMPAR‐Mediated Excitatory Transmission

**FIGURE 4:**
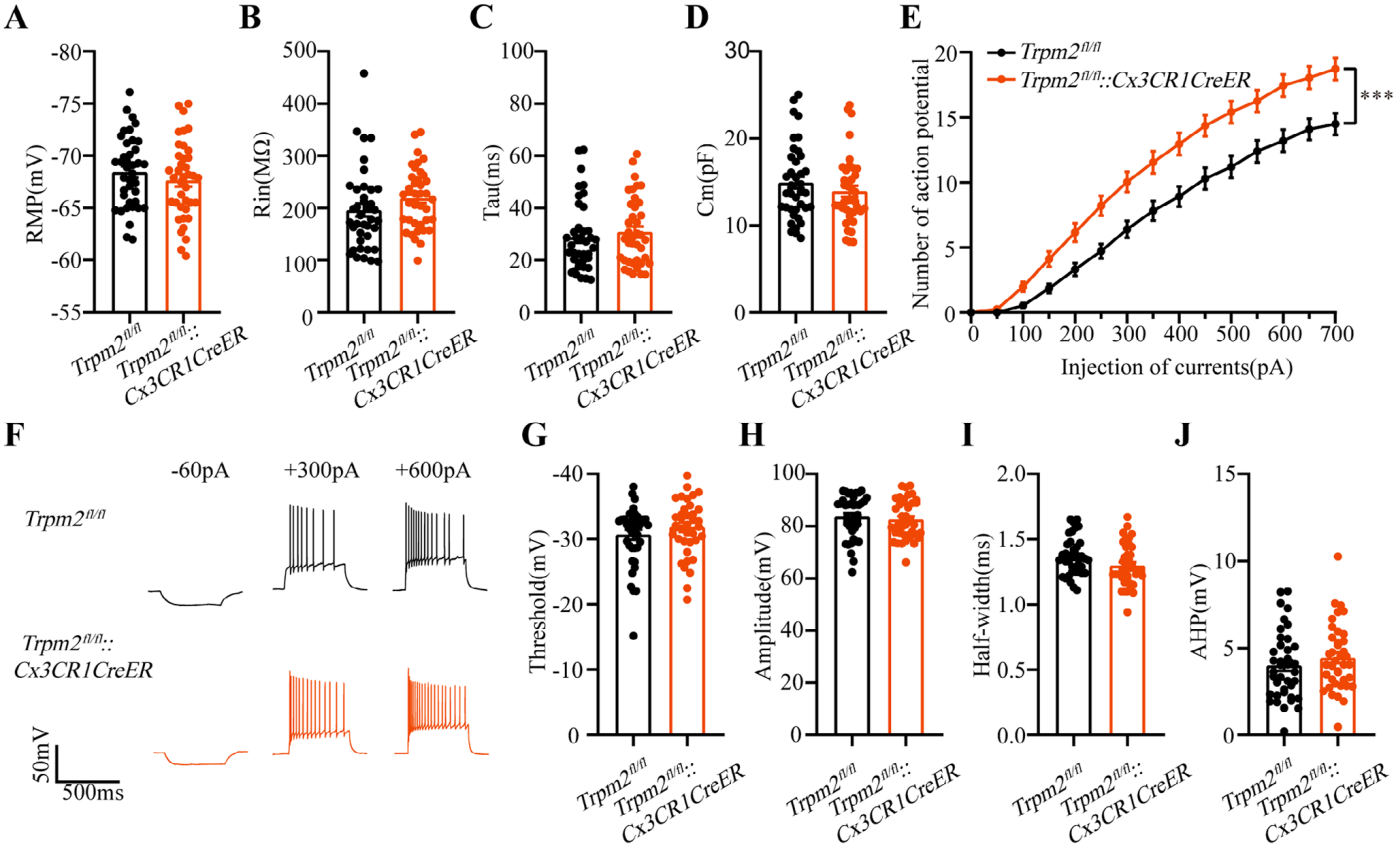
Microglial *Trpm2* deletion increases the excitability of hippocampal pyramidal neurons. (A–E) The effect of selective knockout of TRPM2 channel in microglia on RMP (A), Rin (B), Tau (C), Cm (D), and curve of current‐action potentials (E) of hippocampal pyramidal neurons of mice. (F) Representative action potentials in different injected currents. (G–J) Threshold (G), amplitude (H), half‐width (I), and AHP (J) of action potential in *Trpm2^fl/fl^* and *Trpm2^fl/fl^::Cx3CR1CreER* mice (*n* = 39 from 6 mice for each group). Error bars are means ± s.e.m.; two‐tailed unpaired *t*‐test was used in A–D and G–J; two‐way ANOVA test was used in E; *** represents *p* < 0.001.

The neural hyperexcitability is one of main causes of epilepsy [2, 3], and to confirm the role of microglial TRPM2 channel, we evaluated whether the deficiency of TRPM2 channel in microglia is essential to influence the excitability of hippocampal pyramidal neurons. Interestingly, we found that the RMP, Rin, Tau, and Cm of hippocampal neurons had no change after microglial *Trpm2* deletion (Figure 4A–D), but the spike number was increased when injecting the same depolarizing current (Figure 4E,F), showing that microglial *Trpm2* deletion increased the excitability of pyramidal neurons in hippocampus. Furthermore, we analyzed the specific indicators of action potential and then we found that the threshold, amplitude, half‐width, and after hyperpotential (AHP) of action potential had no difference between *Trpm2^fl/fl^* and *Trpm2^fl/fl^::Cx3CR1CreER* mice (Figure 4G–J). Previous study found that the genetic knockout of TRPM2 increased neuronal excitability by inhibiting Kv7 channel [40], and thus we also recorded M currents of Kv7 channel in hippocampal neurons acquired from their descriptive method. The result showed that the M currents were indeed decreased in *Trpm2^fl/fl^::Cx3CR1CreER* mice (Figure S7A,B). To further confirm the influence of microglial *Trpm2* deletion on Kv7 channel, we evaluated the seizure behavior after intraperitoneal injection 10 mg/kg Kv7 activator retigabine, and the mice still developed tonic seizure and the incidence of death was not inhibited (Figure S7E–H), suggesting that the Kv7 channel was not the main direct factor of *Trpm2* deletion on neuronal excitability. The increase of neuronal excitability may due to the increased release of presynaptic glutamate. The paired‐pulse ratio data (Figure S7C) showed no changes after the microglial *Trpm2* deletion, suggesting that presynaptic glutamate release probability remained unchanged.

**FIGURE 5:**
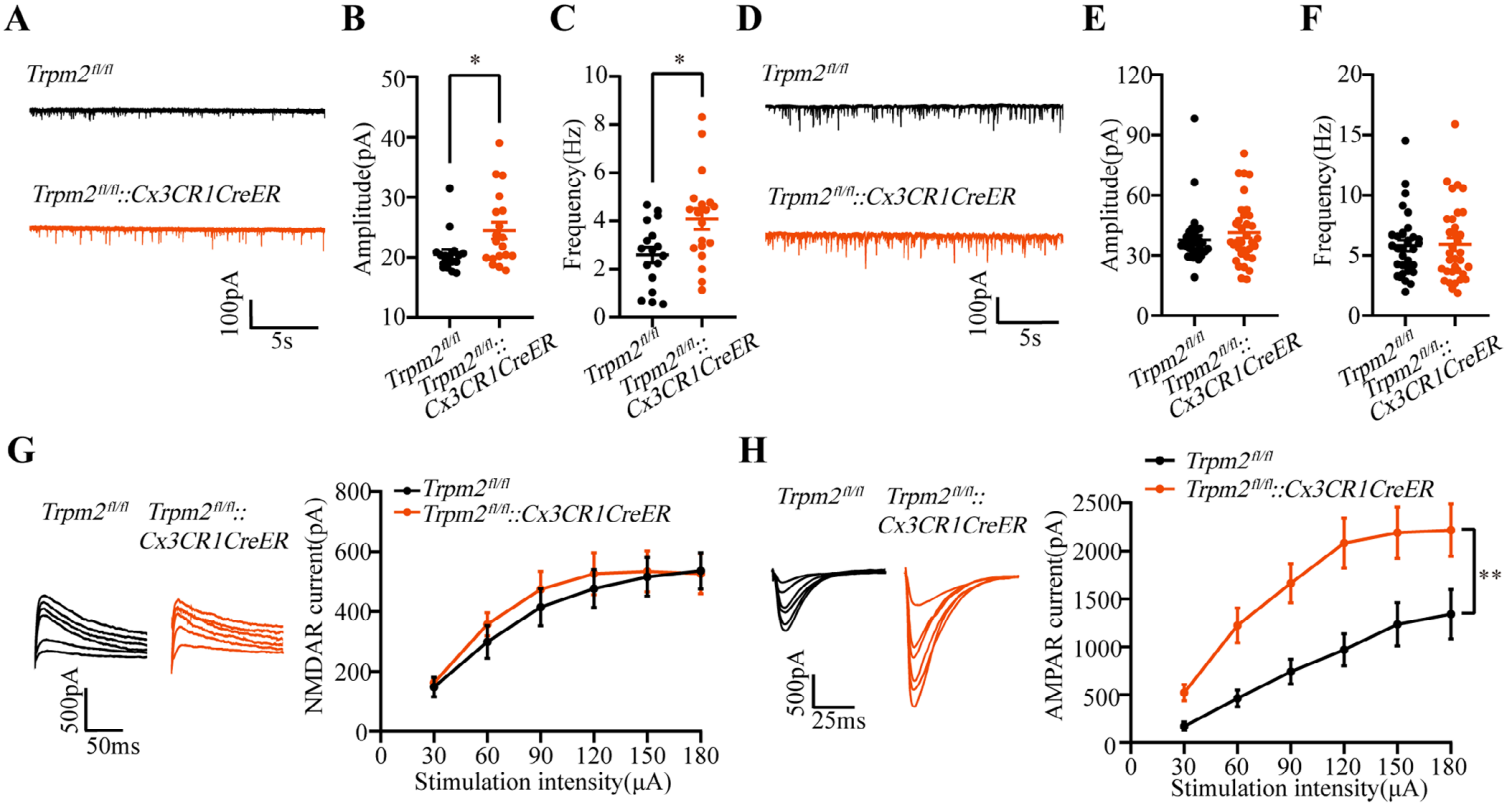
Microglial *Trpm2* deletion increases AMPAR‐mediated excitatory synaptic transmission. (A) Representative traces showing sEPSCs in pyramidal neuron from *Trpm2^fl/fl^* and *Trpm2^fl/fl^::Cx3CR1CreER* mice. (B and C) Amplitude of sEPSCs and frequency of sEPSCs (*n* = 17 from 4 mice for *Trpm2^fl/fl^*, *n* = 19 from five mice for *Trpm2^fl/fl^::Cx3CR1CreER*). (D) Representative traces showing sIPSCs in pyramidal neuron from *Trpm2^fl/fl^* and *Trpm2^fl/fl^::Cx3CR1CreER* mice. (E and F) Amplitude of sIPSCs and frequency of sIPSCs (*n* = 36 from three mice for *Trpm2^fl/fl^*, *n* = 37 from three mice for *Trpm2^fl/fl^::Cx3CR1CreER*). (G) Representative evoked NMDAR current recorded in pyramidal neurons from *Trpm2^fl/fl^* and *Trpm2^fl/fl^::Cx3CR1CreER* mice by electrical stimulation (left). Summary data are shown in right (*n* = 21, 16 from each four mice for *Trpm2^fl/fl^* and *Trpm2^fl/fl^::Cx3CR1CreER*). (H) Representative evoked AMPAR current recorded in pyramidal neurons from *Trpm2^fl/fl^* and *Trpm2^fl/fl^::Cx3CR1CreER* mice by electrical stimulation (left). Summary data are shown in right (*n* = 19, 21 from each four mice for *Trpm2^fl/fl^* and *Trpm2^fl/fl^::Cx3CR1CreER*). Error bars are means ± s.e.m.; two‐tailed unpaired *t*‐test was used in B, E, and F; Mann–Whitney test was used in C; two‐way ANOVA test was used in G and H; * and ** represent *p* < 0.05 and 0.01, respectively.

Then, we hypothesize that this phenomenon may be partly due to the effects of synaptic integration [41]. Thus, we evaluated the postsynaptic transmission, and we found that the amplitude and frequency of sEPSCs were both increased (Figure 5A–C), but the amplitude and frequency of sIPSCs were not changed (Figure 5D,E) in *Trpm2^fl/fl^::Cx3CR1CreER* mice, suggesting microglial TRPM2 channel directly influenced the neural excitatory synaptic transmission to increase neuronal excitability. Furthermore, we revealed the density of synaptic spines in hippocampal CA3 region in *Trpm2^fl/fl^::Cx3CR1CreER* mice by Golgi staining. The data showed that microglial TRPM2 deficiency increased the number of mature synapses in the hippocampal CA3 region but had no effect on synaptic spine density (Figure S7D), suggesting the microglial TRPM2 channel may regulate the neuronal excitability by influencing synaptic structure. Additionally, it is reported that deficiency of TRPM2 channel can cause changes in the expression of excitatory synaptic receptors in neurons [42, 43]. Moreover, knockout of TRPM2 channel can reduce the extrasynaptic NMDA‐induced Ca^2+^ response, but does not affect synaptic NMDARs [27]. Thus, we recorded the evoked NMDAR‐mediated currents and AMPAR‐mediated currents. The results showed that compared with *Trpm2^fl/fl^* mice, the evoked AMPAR‐mediated currents instead of NMDAR‐mediated currents were increased in *Trpm2^fl/fl^::Cx3CR1CreER* mice (Figure 5G,H), providing a possible mechanism explanation for microglial TRPM2 channel increasing the neural excitability to result in epilepsy susceptibility. Then, we also found that the 30 mg/kg AMPAR antagonist NBQX by intraperitoneal injection can inhibit the development of seizure behavior in *Trpm2^fl/fl^::Cx3CR1CreER* mice (Figure S7I–L), further showing the microglial TRPM2 channel was closely association with neuronal AMPAR.

## 3 Discussion

In this study, we found that *Trpm2^−/−^* mice displayed more serious seizure behavior than WT mice across three types of acute seizure model. Specifically, latency to tonic seizure of *Trpm2^−/−^* mice was decreased in PTZ‐induced seizure model; latency to GS and SE of *Trpm2^−/−^* mice were decreased in intrahippocampal KA model; the number of stimulations required to reach stage 4 and fully kindled of *Trpm2^−/‐^* mice was decreased in hippocampal kindling model; the results consistently showed that the deficiency of TRPM2 channel plays a proepileptic role in later stages of seizure development, rather than in initial seizure susceptibility. Previous studies have reported conflicting results regarding the effect of TRPM2 knockout on seizures [40, 44], likely due to differences in models and disease stages. Interestingly, we identified microglial rather than CaMKIIα^+^ or PV^+^ neuronal TRPM2 channel as key regulators in seizure development. Microglial TRPM2 channel appears to modulate neuronal synaptic transmission with independence of neuroinflammation. Indeed, inflammatory cytokines from both neurons and glia cells contribute to epilepsy [45], although the microglial TRPM2 deficiency can inhibit microglial activation, it cannot inhibit the release of inflammatory cytokines in epilepsy (later phase of seizure development), which increased the excitotoxicity and neuronal loss further promoted the release of inflammatory cytokines from all types of neural cells [45]. Moreover, the activation of astrocytes also can increase the release of inflammatory cytokines in epilepsy [46, 47]. Besides, it is reported that noninflammatory changes in microglia are sufficient to induce epilepsy [48]. Therefore, we speculate that the timing of inflammatory factor detection after seizures may explain why we only observed microglial activation without detecting changes in inflammatory factors. Since the available TRPM2 protein antibodies on the market have poor specificity, it is impossible to perform Western Blot validation on mouse brain tissue directly, and it is another promising point to be resolved in future work.

Epilepsy is a prevalent and heterogeneous neurological disorder. The various types of epilepsy patients and animal models, indicating the complex pathophysiology of epilepsy, need different types of antiseizure medication. A study shows that several antiseizure drugs (such as ethosuximide and diazepam) are effective in PTZ model, but not in MES test [49]. As another example, levetiracetam is ineffective in PTZ and MES seizure models, but highly effective in kindling seizure model. Besides, mTOR antagonist rapamycin (RAP) had anticonvulsant effects in PTZ‐ not NMDA‐ and KA‐induced seizure models [50, 51, 52]. Usually, most appropriate treatment may bring the highest chances of seizure freedom and better seizure control. While, we found that microglial TRPM2 channel deficiency mice display a proepileptic behavior independent of the types of seizure models, indicating the generalized role of microglial TRPM2 in seizures development. Our results provide compelling evidence that microglial TRPM2 channel might be promising drug target for the treatment of seizure development. Considering the potential drug targeting on microglial TRPM2 channel, it is necessary to develop precise microglia drug delivery system to exclude the side effects on neurons, liposomes and exosomes are promising solution realizing microglia target [53, 54] to directly regulate the function of microglia. In recent years, research on intracranial drug delivery has been increasing, Gao, Tylawsky, and others have found that utilizing nanoparticle delivery systems and the phagocytic clearance ability of microglia enables targeted drug delivery to specific microenvironments in the brain, thereby reducing drug toxicity [55, 56, 57].

As is well known, TRPM2 channel is abundant in central nervous system and is involved in many physiopathological processes such as synaptic plasticity regulation and neuronal diseases [27, 35, 40, 58]. As a Ca^2+^‐permeable channel, previous studies show that TRPM2 channel is involved in excitotoxicity in ischemic stroke via coupling with synaptic NMDARs and increased endogenous bilirubin can activate TRPM2 channel to aggravate Ca^2+^‐dependent brain injury in stoke patients and mouse models [27, 59], suggesting TRPM2 channel can regulate the excitatory of neurons. However, due to the limitation of the above studies not employing the conditional TRPM2 knockout mice, whether the microglial TRPM2 is responsible for the abnormal neuronal activity in stroke is still unclear. Traditionally, it is considered that epilepsy is mainly caused by hypersynchronous firing of neurons; however, our results revealed that neuronal TRPM2 channel is not involved in later stage of seizure development. According to UMAP plot analysis, TRPM2 channel is more abundant in microglia than other neurons, which may explain the reason of microglial but not neuronal TRPM2 channel plays a vital role in seizure.

The historical focus of epilepsy study has been neurocentric, while emerging evidence have pointed to the role of microglia in modulating seizure. However, the underlying mechanisms of microglia mediating epileptogenesis still remain unclear. Here, we unveil a novel mechanism though which microglial TRPM2 mediates epilepsy development. We have also noticed that TRPM2 channel mediating microglial activation and neuroinflammation imply the molecular mechanisms for AD progression [58]. Accumulating evidence have shown that microglia is involved in epilepsy by synaptic pruning and neuroinflammation regulation [20, 21]. Fractalkine receptor (CX3CR1) regulates microglial recruitment to synapses, which is necessary for synaptic pruning and maturation [60]. Knocking down Acyl‐CoA synthetase long‐chain family member 4 (ACSL4) in microglia decreased proinflammatory cytokine production alleviating neuroinflammation in a systemic LPS model [61]. It is reported that knockout of Trpm2 in microglia attenuates neuroinflammation within the hippocampus during the postinsult latent period in IHKA mice [62]. However, our study found that when tissues were collected immediately after GSs, the levels of inflammatory factors did not change, indicating that TRPM2 in microglia may contribute to epilepsy in a noninflammation‐dependent manner. The different phenomenon may be related to the different stages of epilepsy. It is hypothesized that the activation of microglia is protective in early stage of epilepsy induction, and overactivation of microglia is harmful after the epilepsy. Our study raises the proof of concept that microglia TRPM2 may regulate the epileptogenesis through a noninflammatory‐dependent mechanism.

Recently, a hot topic of research in the field of microglia is that in addition to mediating inflammation, microglia can manipulate neuronal excitability by modulating neuronal synaptic function. For example, mice lacking the chemokine receptor Cx3cr1 exhibit a consequent weak synaptic transmission and conditional deletion the microglia‐specific proteins fractalkine receptor can affect the maturation and synaptic plasticity of neurons [63, 64]. In this study, we used the microglial TRPM2 deficiency mice to determine that microglial TRPM2 deficiency can increase the excitatory of pyramidal neurons in hippocampus. Consistently, accumulating evidence have shown that microglia is involved in epilepsy by synaptic pruning [20, 21]. It is reported that microglia can regulate neuronal AMPAR by influencing the oxidative stress and synaptic pruning of microglia [65, 66]. For example, microglia can regulate AMPAR function of neurons by complement receptor 3 (CR3) activation. In this work, microglial CR3‐triggered LTD results from NADPH oxidase activation and AMPAR internalization [66]. Similarly, our electrophysiological results showed that microglial *Trpm2* deletion increased the excitatory postsynaptic currents and AMPAR‐mediated currents of pyramidal neurons. Ionotropic glutamate receptors are critical for neuronal excitation/inhibition balance and glutamate signaling transmission exhibits dysfunction in epilepsy. Also, transcriptomic analysis revealed that a significant increase of genes coding for AMPA receptor and hyperexcitation of neurons due to the increased AMPAR expression in seizure [67, 68, 69]. In addition, previous studies have found that TRPM2 channel can promote the trafficking of extrasynaptic NMDA receptor in ischemic models to induce excitotoxicity. Our findings showed that microglia TRPM2 channel is also able to regulate neuronal AMPA receptor activity through cell–cell crosstalk. Since the precise mechanism remains elusive, it will be interesting to explore how microglial TRPM2 channel regulate the AMPA receptor of neurons in future.

## 4 Materials and Methods

### 4.1 Animals

*Trpm2^−/−^* mice on the C57BL/6 background were generated as previously described [25]. *Trpm2^fl/fl^* mice were generated via standard homologous recombination, with exon 4 flanked by loxP sequences. To achieve cell type‐specific deletion of *Trpm2* in CaMKIIα⁺ excitatory neurons, PV⁺ interneurons, and microglia, *Trpm2^fl/fl^* mice were crossed with *CaMKIIα‐Cre* mice (Jax No. 005359), *PV‐Cre* mice (Jax No. 008069), or *Cx3CR1CreER* mice (Jax No. 021160), respectively. 120 mg/kg tamoxifen dissolved in corn oil 1 day for each mouse for 5 day was injected to induce expression of Cre‐recombinase in *Trpm2^fl/fl^::Cx3CR1CreER* mice. Male mice aged 6–12 weeks were used in experiments. Mice were housed under a 12 h light/dark cycle (lights on 8:00 a.m.–8:00 p.m.) at 21–23°C and 40–50% humidity, with free access to food and water. All animal procedures were approved by the Animal Advisory Committee of Zhejiang University (Approval No. 14973) and conducted in accordance with both its guidelines and the NIH Guidelines for the Care and Use of Laboratory Animals.

The primers are Trpm2–floxp F: GATTAAGCACAGGATGGGCT; Trpm2–floxp R: ACAAGGATGAGCCTGTGTGA. For the pair of Trpm2–floxp F and Trpm2–floxp R, the PCR products of floxed allele were 470 bp and the wild‐type allele were 350 bp.

### 4.2 Stereotactic Surgery

Mice were anesthetized with Na^+^ pentobarbital (50 mg/kg, i.p.) and secured in a stereotaxic frame (68526; RWD life Science Co., Ltd, China). After exposing the skull, burr holes were drilled stereotactically. A cannula (62003; RWD Life Science Co., Ltd) was implanted into the right dorsal hippocampus (AP: −2.0 mm; ML: −1.3 mm; V: −1.6 mm) for drug delivery, and an electrode (95500, 0.125 mm diameter; A.M. Systems) was placed in the right ventral hippocampus (AP: −2.9 mm; ML: −3.2 mm; V: −3.2 mm) for EEG recording [70]. Coordinates were based on the Franklin and Paxinos [71].

### 4.3 PTZ‐Induced Seizure Model

Mice were treated with PTZ (60 mg/kg, i.p) to induce seizure similar as our previous study [72, 73]. EEG activity was recorded using a LabChart system (AD Instruments) as mice were placed in PVC boxes immediately after PTZ injection. Behavior was monitored for 30 min, and seizure severity was scored: 1, facial movements; 2, head nodding; 3, forelimb clonus; 4, rearing with clonus; 5, rearing and falling with clonus; 6, full tonic seizure (sudden muscle stiffness). Scores 4–6 were classified as GSs [74]. For each mouse, seizure stage, onset of EEG seizure, latency to GS, latency to tonic seizure, and incidence of death were recorded. If GS or tonic seizure did not occur within 30 min, latency was recorded as 30 min.

### 4.4 Intrahippocampal KA Model

As mentioned above, a cannula and an electrode were implanted into right dorsal and ventral hippocampus for kainic acid (KA) delivery and EEG recording respectively. After 1‐week surgery recovery, KA (0.25 mg in 0.5 mL saline) was injected into dorsal CA1 through cannula over 2 min with a 1 mL microsyringe. Behavior was monitored for 90 min and seizure severity was scored [75]. For each mouse, the highest seizure stage within each 10 min, the highest seizure stage within 90 min, onset of EEG seizure, latency to GS, number of GS, and latency to SE were recorded. If GS or tonic seizure did not occur within 30 min, latency was recorded as 90 min.

### 4.5 Hippocampal Kindling Model

As mentioned above, a twin bipolar electrode was implanted into the right ventral hippocampus for kindling stimulation and EEG recording. After 1‐week surgery recovery, the ADT was determined for each mouse using monophasic square‐wave pulses(20 Hz, 1 ms/pulse, 40 pulses) delivered via a constant‐current stimulator (SEN‐7203, SS‐202J; Nihon Kohden). EEGs were recorded using a Neuroscan system (NuAmps, Neuroscan System) as our previous studies [34, 75]. Stimulation began at 40 µA and increased by 20‐µA every minute until a ≥5 s ADD was observed; this intensity was defined as the ADT. Mice were then subjected to 10 daily kindling stimulations (400 µA, 20 Hz, 2 s trains, 1 ms pulses) as our previous study [34]. Seizure severity was assessed according to the Racine scale: 1, facial movement; 2, head nodding; 3, unilateral forelimb clonus; 4, bilateral forelimb clonus and rearing; 5, rearing and falling. Stages 1–3 were considered]focal seizures and stages 4–5 as GSs [76]. Mice were deemed fully kindled after three consecutive stage 5 seizures. Seizure stage and ADD were recorded for each stimulation, along with the number of stimulations required to reach stages 2, 4, and full kindling.

### 4.6 Reanalysis of snRNA‐seq Data

SnRNA‐seq data from healthy frozen human postmortem midbrain tissue (GEO: GSE157783) were retrieved from GEO. A filtered feature‐barcode matrix was used for analysis in Seurat v4.0.1. Cells with <150 genes or >20% mitochondrial RNA were excluded. UMAP was used for dimensionality reduction (top 20 PCs), and cells were clustered with FindClusters (resolution = 0.6). Marker genes were identified using FindAllMarkers (avg_log2FC.threshold = 0.3, test.use = “wilcox”). Clusters were annotated based on canonical markers.

### 4.7 In Vitro Electrophysiology

The brain was removed quickly and immersed in ice‐cold artificial cerebrospinal fluid (ACSF) (in mM: 120 NaCl, 11 dextrose, 2.5 KCl, 1.28 MgSO_4_, 3.3 CaCl_2_, 1 NaH_2_PO_4_, and 14.3 NaHCO_3_, bubbled with 95% O_2_ and 5% CO_2_. Coronal slices (300 µm) were cut using a vibratome (VT1000 mol/L/E, Leica) and incubated at 25°C for 1 h. Slices were transferred to a recording chamber for patch clamp recording. Patch pipette (5–10 MΩ resistance) was filled with recording solution (in mM: 140 K‐gluconate, 5 NaCl, 0.2 EGTA, 2 Mg‐ATP, 10 HEPES) and placed in CA3 region for recording after compensating. Signals were amplified and recorded using a MultiClamp 700B amplifier (Axon Instruments).

To test action potential threshold, episodic currents were injected in 50 pA steps (0–700 pA). To record spontaneous synaptic currents, low‐divalent ion ACSF was used (in mM: 125 NaCl, 3.5 KCl, 1.25 NaH_2_PO_4_, 0.5 MgCl_2_, 26 NaHCO_3_, 25 dextrose, and 1 CaCl_2_). Pipettes filled with cesium‐based internal fluid (in mM: 100 CsCH_3_SO_3_, 20 KCl, 10 HEPES, 4 Mg‐ATP, 0.3 Tris‐GTP, 7 Tris2‐phosphocreatine, and 3 QX‐314) were used to record sEPSCs at −70 mV with 20 µM bicuculline. Similarly, pipettes filled with cesium‐based internal fluid (in mM: 120 CsCl_2_, 5 NaCl, 10 HEPES, 1 MgCl_2_, 3 Mg‐ATP, 0.3 Na_2_‐GTP, 10 EGTA, and 5 QX‐314) were used to recorded sIPSCs at −70 mV with 10 µM CNQX. Events were analyzed with MiniAnalysis software (version 6.0.3).

For evoked EPSC recordings, internal solution (in mM: 125 CsMeSO_4_, 20 CsCl_2_, 10 NaCl, 2 Mg‐ATP, 10 HEPES, 0.2 EGTA, 0.3 Na_3_GTP, 2.5 QX314, pH 7.3 adjusted with CsOH) was used. Neurons were held at −70 mV to record AMPAR‐mediated EPSCs and at +40 mV to record NMDAR‐mediated EPSCs. Isolated NMDAR currents were recorded by adding 20 µM bicuculline and 10 µM CNQX to ACSF, while isolated AMPAR currents were recorded with 20 µM bicuculline and 30 µM DL‐AP5.

### 4.8 Statistical Analysis

Number of experimental replicates (*n*) is indicated in figure legend. Gaussian distribution was tested using the Shapiro–Wilk test. Student's *t*‐test was applied for two normally distributed datasets. ANOVA was used for comparisons of more than two datasets. For non‐normal data, nonparametric tests were employed: Mann–Whitney *U* test for single comparisons, Kruskal–Wallis test for one‐way analysis. Statistical analysis was performed using Prism (8.0) or SPSS (19.0), with significance set at *p < 0.05, **p < 0.01, and ***p < 0.001.

Further details on materials and methods can be found in the Supporting Information.

## Author Contributions

Y.W., Z.C., and W.Y. conceptualized the main idea of this study. Y.W.X. and L.Y.Y. performed the experiments and conducted data analysis. Z.S.L. and Y.Z. contributed to the identification of transgenic animals. N.H. was responsible for the analysis of the snRNA‐seq data. X.J.W. carried out the Golgi staining. W.J.L.L. and J.X. contributed to the seizure behavioral testing. L.Y.C., C.L.X., J.J.F., J.H.L., L.H.J., and F.H. provided technical guidance and participated in data discussion. Y.W.X. and Y.W. drafted the manuscript. Y.W., Z.C., and W.Y. supervised all aspects of the work. All authors have read and approved the final manuscript.

## Conflicts of Interest

The authors declare no conflicts of interest.

## Ethics Statement

Ethics approval for this study was obtained from the National Natural Science Foundation of China (No. 14973).

## Supporting information




**Supporting file 1**: mco270271‐sup‐0001‐SuppMat.docx

## Data Availability

The data that support the findings of this study are included in the Supporting Information.
