# Supplementary material for: Microglial Transient Receptor Potential Melastatin 2 Deficiency Accelerates Seizure Development via Increasing AMPAR‐Mediated Neuronal Excitability
Source: MedComm (2020). 2025 Jul 14;6(8):e70271. doi: 10.1002/mco2.70271 (PMC12260126; doi:10.1002/mco2.70271)
Supplement: Supplementary file 1 — Supporting file 1: mco270271‐sup‐0001‐SuppMat.docx [file MCO2-6-e70271-s001.docx]

**Microglial Transient receptor potential melastatin 2 Deficiency Accelerates Seizure Development Via Increasing AMPAR-mediated Neuronal Excitability**

Yingwei Xu^1,2#^, Luyu Ye^1,3#^, Zhisheng Li^1^, Yi Zhang^1,3^, Ning Hua^1,3^, Xiaojun Wang^4^, Wangjialu Lu^1^, Jing Xi^1^, Liying Chen^1^, Cenglin Xu^2^, Jiajia Fang^3^, Jianhong Luo^4^, Linhua Jiang^5^, Feng Han^6^, Zhong Chen^1,2*^, Yi Wang^1,2,3*^, Wei Yang^1,3*^

1 Institute of Pharmacology & Toxicology, NHC and CAMS Key Laboratory of Medical Neurobiology, College of Pharmaceutical Sciences, School of Medicine, Zhejiang University, Hangzhou, China.

2 Zhejiang Key Laboratory of Neuropsychopharmacology, School of Pharmaceutical Sciences, Zhejiang Chinese Medical University, Hangzhou, China.

3 Department of Biophysics and Department of Neurology, the Fourth Affiliated Hospital Zhejiang University School of Medicine, Yiwu, China.

4 Department of Neurobiology, Affiliated Mental Health Center, College of Brain Science and Brain Medicine, School of Medicine, Zhejiang University, Hangzhou, China

5 Sino-UK Joint Laboratory of Brain Function and Injury of Henan Province and Department of Physiology and Pathophysiology, Xinxiang Medical University, Xinxiang, China

6 International Joint Laboratory for Drug Target of Critical Illnesses, School of Pharmacy, Nanjing Medical University, Nanjing, China.

**# Yingwei Xu and Luyu Ye contributed equally to this work**

*** Corresponding authors:**

Prof. Wei Yang, PhD., [yangwei@zju.edu.cn](mailto:yangwei@zju.edu.cn),

Prof. Yi Wang, PhD., [wang-yi@zju.edu.cn](mailto:wang-yi@zju.edu.cn),

Prof. Zhong Chen, PhD., [chenzhong@zju.edu.cn](mailto:chenzhong@zju.edu.cn)

**Supplementary materials and methods**

**Viral vectors and injection**

AAV2/9-mCaMKIIa-H2B-eGFP-P2A-iCre-WPRE-pA (viral titers:1.9 × 1013 v.g./mL), AAV2/9-mCaMKIIa-H2B-eGFP-WPRE-pA (viral titers: 1.72 × 1013 v.g./mL) were produced by Taitool Bioscience Co., Ltd (Shanghai, China). rAAV-PV-CRE-EGFP-bGH polyA (viral titers: 3.01 × 1012 v.g./mL), rAAV-PV-EGFP-bGH polyA (viral titers: 5.42 × 1012 v.g./mL) were produced by BrainTVA Co., Ltd (Wuhan, China). All vectors were stored at −80 °C before use. Mice received 200 nL of virus bilaterally into the ventral hippocampus (AP: −2.9 mm, ML: ±3.2 mm, DV: −3.2 mm) or SNr (AP: −3.2 mm, ML: ±1.2 mm, DV: −4.5 mm) at 40 nL/min using a microsyringe pump(Micro4, World Precision Instruments). Behavioral testing was conducted at least 3 weeks post-injection.

**MES-induced seizure model**

Maximal electroshock seizures (MES) were induced using ear-clip electrodes delivering 6 mA at 50 Hz for 0.2 s via a rodent shocker (Hugo Sachs Elektronik, March-Hugstetten, Germany). Tonic hindlimb extension indicated successful seizure induction. If no response occurred, current was increased by 1 mA after 2 minutes. Seizure threshold, tonic duration, and mortality were recorded.

**Immunohistochemistry and Golgi Staining**

Following perfusion with PBS and 4% PFA, brains were cryosectioned (30 μm) in a cryostat (NX70, Thermo). Sections were stained with anti-Iba1 antibody (goat, 1:500, ab5076, abcam) and Alexa 488 secondary antibody, then imaged using a Leica SP8 confocal microscope. Golgi staining (FD Rapid Golgi Kit) was performed on 150 μm coronal slices. Dendritic spine morphology was analyzed in CA3 pyramidal neurons using an Olympus BX61 microscope. ImageJ was used for 3D reconstruction and quantification.

**Cell culture and TRPM2 current recording**

HEK293 cells transfected with wild-type and exon 4-deleted TRPM2(m) plasmids. Transient transfections were carried out using Lipofectamine 3000 Transfection Reagent (Invitrogen, USA) according to the manufacturer's protocol. Cells were used for electrophysiological experiments 24 h after transfection. The currents were recorded by applications of a 500 ms voltage ramp from −100 to + 100 mV every 5 s, with a holding potential of −40 mV.

**Microglia isolation**

Microglia were isolated from adult mouse brains using papain digestion and CD11b magnetic beads. Total RNA from hippocampus was extracted using the RNeasy Kit. Real-time qPCR was performed with SYBR Green reagents and normalized to Actin using the ΔΔCT method. The forward and reverse primer sequences used were as follows: 5’-GGCTGTATTCCCCTCCATCG-3’, 5’-CCAGTTGGTAACAATGCCATGT-3’ for Actin; 5’-CTCCGACGAAGCAATAGCAG -3’, 5’-TCTCGGGAATCCATGAGCTAAG -3’ for TRPM2; 5’-TGGCAACTGTTCCTGAACTC-3’, 5’-GGAAGCAGCCCTTCATCTTT-3’ for IL-1β; 5’-TACCACTTCACAAGTCGGAGG-3’, 5’-CTGCAAGTGCATCATCGTTGTT-3’ for IL-6; 5’-GCCTCTTCTCATTCCTGCTT-3’, 5’-TGGGAACTTCTCATCCCTTTG-3’ for TNFα; 5’-AAAGACAATCAGGCCATCAG-3’, 5’-TGGGTTGTTGACCTCAAACT-3’ for IFNγ; 5’-CCCCAGCTAGTTGTCATCCTG-3’, 5’-CAAGTGATTTTTGTCGCATCCG-3’ for IL-4; 5’-AGGCGCTGTCATCGATTT-3’, 5’-CACCTTGGTCTTGGAGCTTAT-3’ for IL-10; 5’-CCACCTGCAAGACCATCGAC-3’, 5’- CTGGCGAGCCTTAGTTTGGAC-3’ for TGFβ; 5’-CTCCAAGCCAAAGTCCTTAGAG-3’, 5’-AGGAGCTGTCATTAGGGACATC-3’ for Arg1.

**In vitro electrophysiology**

For Kv7 current (M current) measurement, pipettes filled with an internal solution (mM:120 K-gluconate, 15KCl, 10 HEPES, 4 Mg-ATP, 0.3 Tris-GTP, and 0.5 EGTA) were used, and a voltage step protocol from -20 mV to -50 mV with tetrodotoxin (0.5 μM) and DNQX (10 μM) was added in ACSF to block sodium current and non-NMDA receptor mediated responses, respectively. For paired-pulse ratio recordings, stimuli were set at 2-3 times the threshold current, with a 50 ms interval. The recording electrode was placed in the CA3 pyramidal layer, ~50 μm from the stimulating electrode in the stratum lucidum of CA3, with responses evoked through an Iso-Flex isolator (A.M.P.I.).

**Supplementary Figures**


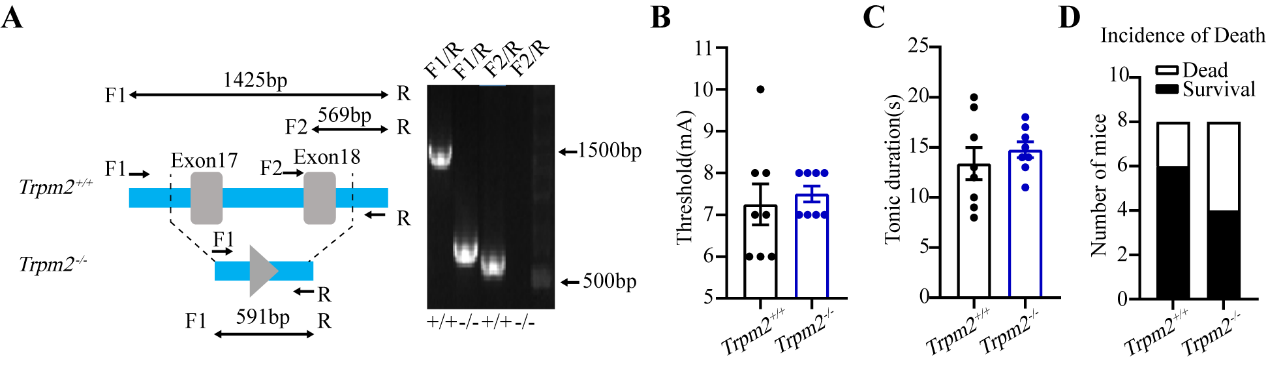


**Figure S1 TRPM2 deficiency has no influence in seizure in MES-induced acute seizure model.**

(A) Schema of Trpm2 gene for knockout (left); PCR analysis disruption of the Trpm2 gene in mouse genome (right). (B-D) The effect of TRPM2 channel knockout on threshold (B), duration (C), incidence of death (D) in MES-induce seizure model (n = 8 for each group). Mann-Whitney test was used for *Trpm2^+/+^* vs *Trpm2^-/-^* in B, C; Chi-square test was used for *Trpm2^+/+^* vs *Trpm2^-/-^* in D.


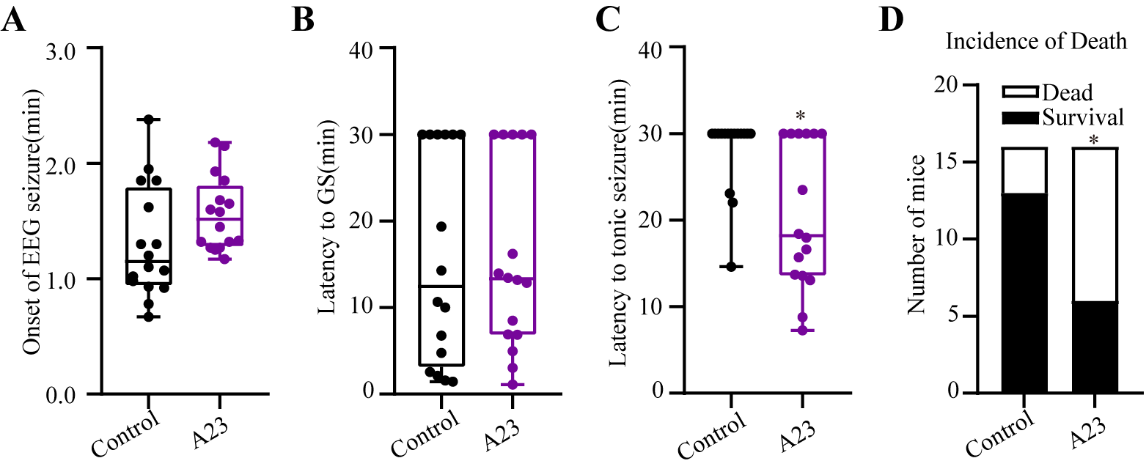


**Figure S2 Pharmacological inhibition of TRPM2 channel by antagonist accelerates seizure in mice.**

(A-D) The effect of TRPM2 channel antagonist on onset of EEG seizure (A), latency to GS (B), latency to tonic seizure (C) and incidence of death (D) in PTZ-induced seizure model (n = 16 for each group). Error bars are means ± s.e.m.; Two-tailed unpaired t-test was used in A; Mann-Whitney test was used in B, C; Chi-square test was used in D; * represent P < 0.05.


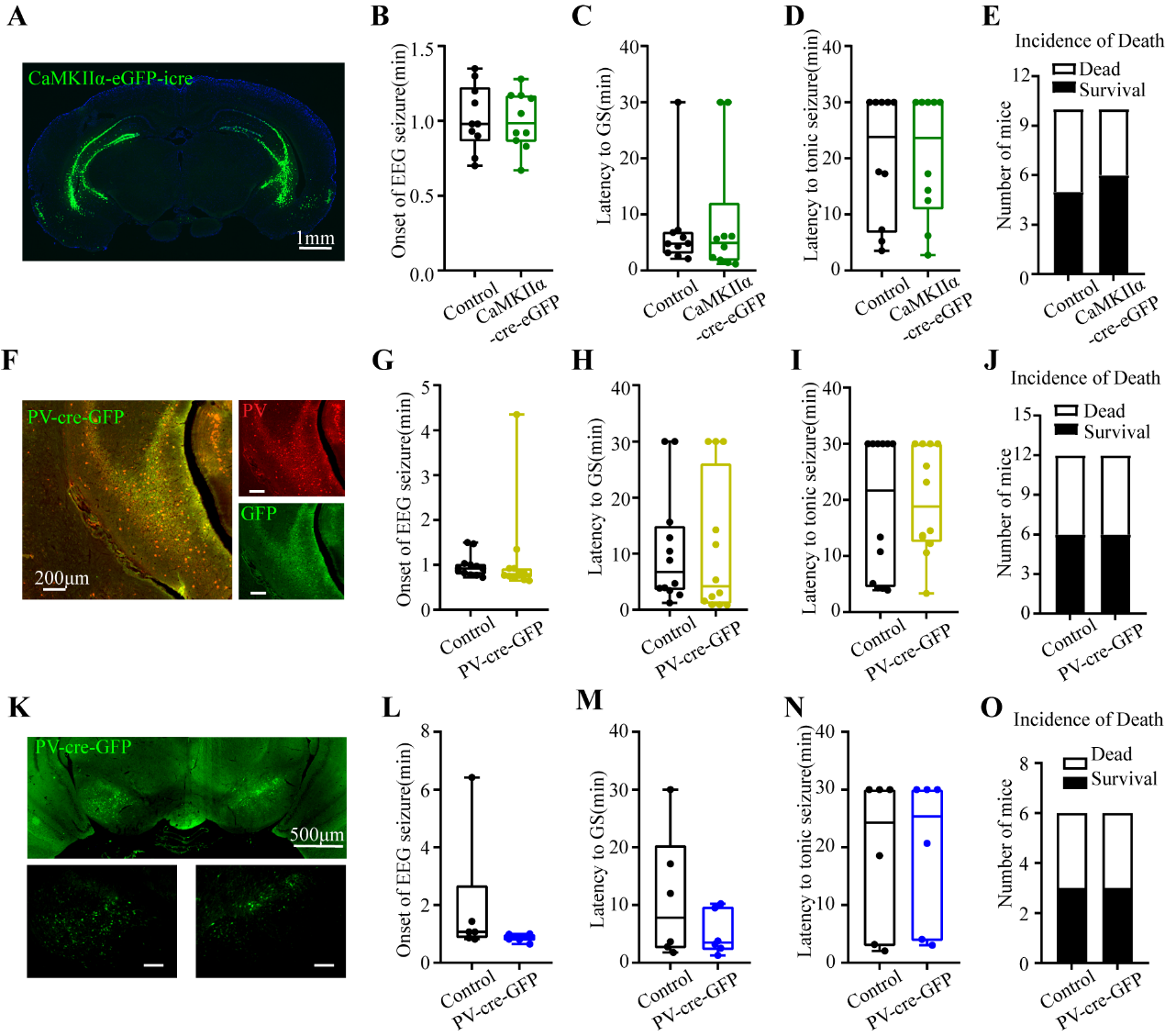


**Figure S3 Selective deficiency of TRPM2 channel in CaMKⅡα^+^ and PV^+^ neurons by virus injection have no influence in seizure susceptibility.**

(A) Injection of virus AAV2/9-mCaMKIIa-H2B-eGFP-P2A-iCre-WPRE-pA or AAV2/9-mCaMKIIa-H2B-eGFP-WPRE-pA in bilateral hippocampus of *Trpm2^fl/fl^* mice. (B-E) Effects of knockout of TRPM2 channel in hippocampal CaMKⅡα**^+^** neurons on onset of EEG seizure (B), latency to GS (C), latency to tonic seizure (D) and incidence of death (E) in PTZ-induced seizure model (n = 10 for control and CaMKIIα-cre-eGFP). (F) Injection of virus rAAV-PV-CRE-EGFP-bGH polyA or rAAV-PV-EGFP-bGH polyA in bilateral hippocampus of *Trpm2^fl/fl^* mice. (G-J) Effects of selective knockout of TRPM2 channel in PV**^+^** neurons on onset of EEG seizure (G), latency to GS (H), latency to tonic seizure (I) and incidence of death (J) in PTZ-induced seizure model (*n* = 15 for each group). (K) Injection of virus rAAV-PV-CRE-EGFP-bGH polyA or rAAV-PV-EGFP-bGH polyA in bilateral SNr of *Trpm2^fl/fl^* mice. (L-O) Effects of selective knockout of TRPM2 channel in PV neurons on onset of EEG seizure (L), latency to GS (M), latency to tonic seizure (N) and incidence of death (O) in PTZ-induced seizure model (*n* = 6 for each group). Error bars are means ± s.e.m.; Two-tailed unpaired t-test was used in B, G, L; Mann-Whitney test was used in C, D, H, I, M, N; Chi-square test was used in E, K, J, O.


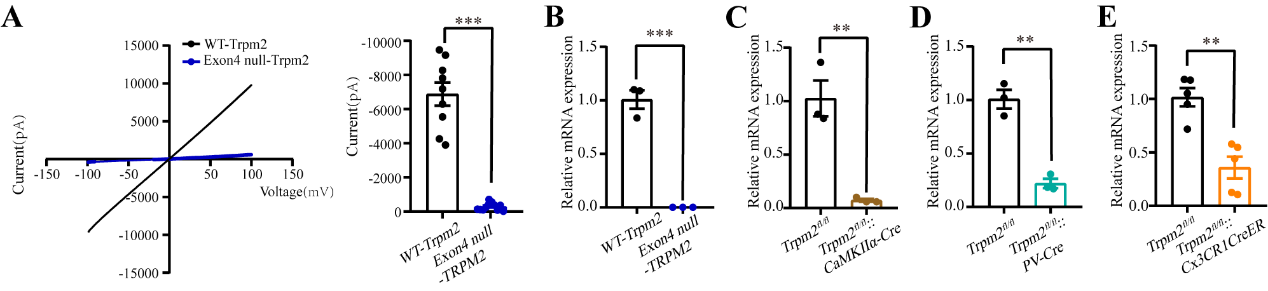


**Figure S4 The relative mRNA expression of TRPM2 was decreased in different conditional knockout mice.**

(A) The TRPM2 channel I-V curve(left) and currents(right) in HEK293 cells transfected with wild-type (WT-trpm2) and exon 4 deleted (Exon4 null-Trpm2) TRPM2(m) plasmids (n = 9, 12 for Wt-trpm2 and exon 4 null-trpm2). (B) The relative mRNA expression in HEK 293 cells (n = 3 for each cell population). (C) The relative mRNA expression in neurons of hippocampus (n = 3 for each mouse). (D) The relative mRNA expression in neurons of SNR (n = 3 for each mouse). (E) The relative mRNA expression in microglia of brain (n = 5 for each mouse). Error bars are means ± s.e.m.; Two-tailed unpaired t-test was used in A-E, **represent P < 0.01, *** represent P < 0.001.

**
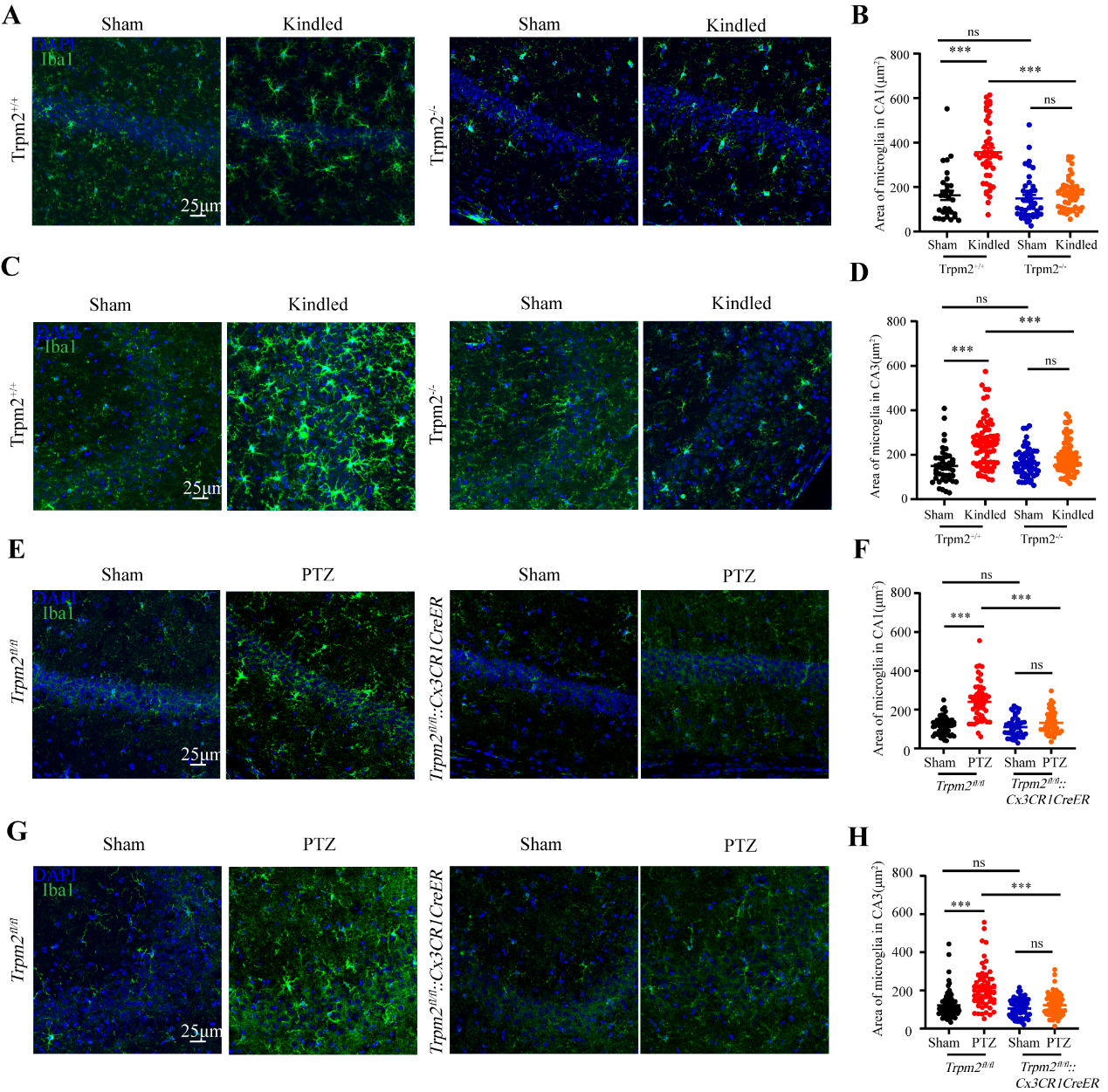
**

**Figure S5 TRPM2 deficiency attenuates morphological activation of hippocampal microglia**

(A) Representative vision of Iba1 and DAPI in CA1 before and after hippocampal kindling. (B) The effect of knockout of TRPM2 channel on the area of microglia in CA1 (*n* = 30, 46, 41, 47 from each 3 mice for *Trpm2^+/+^*-sham, *Trpm2^+/+^*-kindled, *Trpm2^-/-^*-sham and *Trpm2^-/-^*-kindled). (C) Representative vision of Iba1 and DAPI in CA3 before and after hippocampal kindling. (D) The effect of knockout of TRPM2 channel on the area of microglia in CA3 (*n* = 44, 76, 57, 78 from each 3 mice for *Trpm2^+/+^*-sham, *Trpm2^+/+^*-kindled, *Trpm2^-/-^*-sham and *Trpm2^-/-^*-kindled). (E) Representative vision of Iba1 and DAPI in CA1 before and after PTZ-induced seizure. (F) The effect of microglial *Trpm2* deletion on the area of microglia in CA1 (*n* = 57, 41 from each 5 mice for *Trpm2^fl/fl^*-sham, *Trpm2^fl/fl^::Cx3CR1CreER-*sham, *n* = 57, 48 from each 4 mice for *Trpm2^fl/fl^*-PTZ, *Trpm2^fl/fl^::Cx3CR1CreER-*PTZ). (G) Representative vision of Iba1 and DAPI in CA3 before and after PTZ-induced seizure. (H) The effect of microglial *Trpm2* deletion on the area of microglia in CA3 *n* = 76, 57 from each 5 mice for *Trpm2^fl/fl^*-sham, *Trpm2^fl/fl^::Cx3CR1CreER-*sham, *n* = 62, 70 from each 4 mice for *Trpm2^fl/fl^*-PTZ, *Trpm2^fl/fl^::Cx3CR1CreER-*PTZ) .Error bars are means ± s.e.m.; one-way ANOVA test was used in B, D, F, H. *** represent *P* < 0.001.


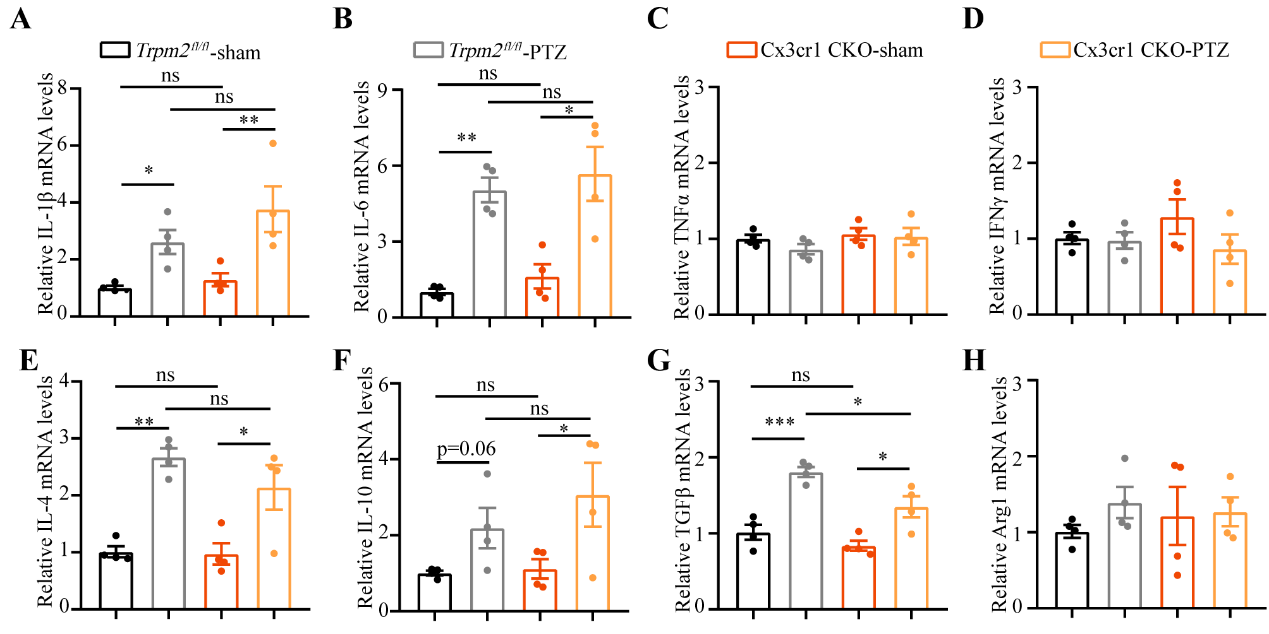


**Figure S6 TRPM2 deficiency has no influence on the expression of inflammation cytokines in epilepsy.**

(A-H) Effects of selective knockout of TRPM2 channel in microglia on the relative mRNA levels of IL-1β (A), IL-6 (B), TNFα (C), IFNγ (D), IL-4 (E), IL-10 (F), TGFβ (G), Arg1 (H) (*n* = 4 for each group). Error bars are means ± s.e.m.; one-way ANOVA test was used in A-H. *, ** and *** represent *P* < 0.05, 0.01 and 0.001, respectively.


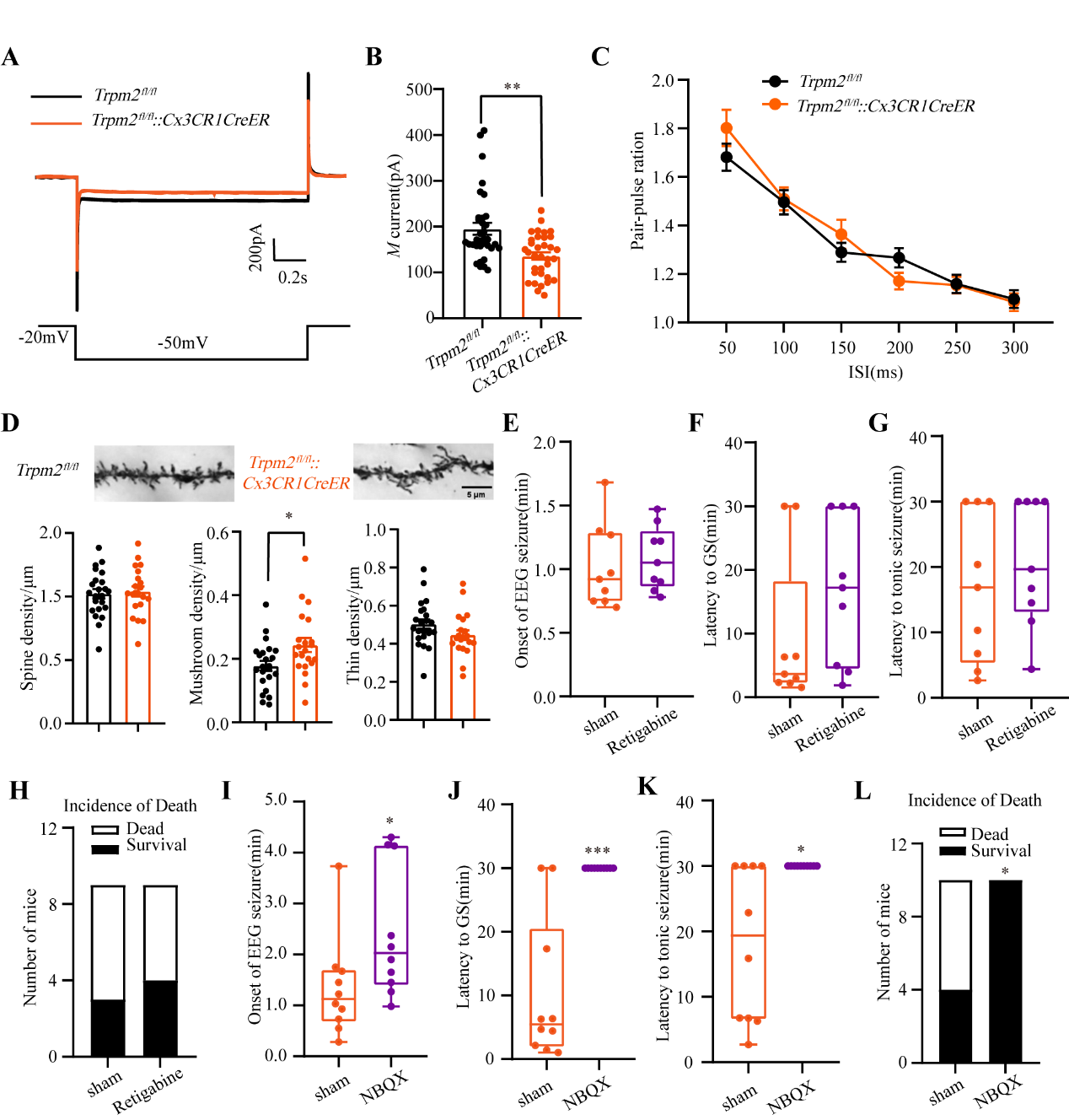


**Figure S7 The role of microglial Trpm2 deficiency in Kv7 current, PPR ration and synaptic density**

(A) Representative traces showing *M* current in pyramidal neuron from *Trpm2^fl/fl^* and *Trpm2^fl/fl^::Cx3CR1CreER* mice. (B) Amplitude of *M* current (n = 34 from 4 mice for *Trpm2^fl/fl^* and *Trpm2^fl/fl^::Cx3CR1CreER* mice). (C) Pair-pulse ration of hippocampal pyramidal neuron from *Trpm2^fl/fl^* and *Trpm2^fl/fl^::Cx3CR1CreER* mice ((*n* = 31 from 5 mice for *Trpm2^fl/fl^*, *n* = 27 from 4 mice for *Trpm2^fl/fl^::Cx3CR1CreER* ).(D) Representative Golgi staining of pyramidal neurons from *Trpm2^fl/fl^* and *Trpm2^fl/fl^::Cx3CR1CreER* mice(upper). Summary data of spine density, mushroom density and thin density are shown in bottom (*n* = 23, 21 from each 3 mice for *Trpm2^fl/fl^* and *Trpm2^fl/fl^::Cx3CR1CreER*). (E-H) The effect of Kv7 activator on onset of EEG seizure (E), latency to GS (F), latency to tonic seizure (G) and incidence of death (H) in *Trpm2^fl/fl^::Cx3CR1CreER* mice in PTZ-induced chemoconvulsion model (n = 9 for each group). (I-L) The effect of AMPAR antagonist on onset of EEG seizure (I), latency to GS (J), latency to tonic seizure (K) and incidence of death (L) in *Trpm2^fl/fl^::Cx3CR1CreER* mice in PTZ-induced chemoconvulsion model (n = 10 for each group). Error bars are means ± s.e.m.; Two-tailed unpaired t-test was used in A, D, E, I; Two-way ANOVA test was used in C; Mann-Whitney test was used in F, G, J, K; Chi-square test was used in H, L; *, **, ***represent P <0.05, 0.01,0.001, respectively.
